# Targeting mitochondrial oxidative stress with MitoQ reduces NET formation and kidney disease in lupus-prone MRL-*lpr* mice

**DOI:** 10.1136/lupus-2020-000387

**Published:** 2020-04-16

**Authors:** Karen A Fortner, Luz P Blanco, Iwona Buskiewicz, Nick Huang, Pamela C Gibson, Deborah L Cook, Hege L Pedersen, Peter S T Yuen, Michael P Murphy, Andras Perl, Mariana J Kaplan, Ralph C Budd

**Affiliations:** 1 Vermont Center for Immunology and Infectious Diseases, Department of Medicine, University of Vermont Larner College of Medicine, Burlington, VT, USA; 2 Systemic Autoimmunity Branch, National Institute of Arthritis and Musculoskeletal and Skin Diseases, National Institutes of Health (NIH), Bethesda, MD, USA; 3 Department of Microbiology and Immunology, Upstate Medical University, Syracuse, NY, New York; 4 Rheumatology Clinic, Upstate University Hospital, Syracuse, NY, New York; 5 Department of Pathology and Laboratory Medicine, University of Vermont Larner College of Medicine, Burlington, VT, USA; 6 Renal Diagnostics and Therapeutic Unit, Kidney Diseases Branch, National Institutes of Diabetes and Digestive and Kidney Diseases, Bethesda, Maryland, United States; 7 MRC Mitochondrial Biology Unit, Biomedical Campus, University of Cambridge, Cambridge, CB2 0XY, UK; 8 Department of Medicine, University of Cambridge, Cambridge, CB2 0QQ, UK

**Keywords:** autoimmune diseases, systemic lupus erythematosus, T cells, inflammation

## Abstract

**Objectives:**

Recent investigations in humans and mouse models with lupus have revealed evidence of mitochondrial dysfunction and production of mitochondrial reactive oxygen species (mROS) in T cells and neutrophils. This can provoke numerous cellular changes including oxidation of nucleic acids, proteins, lipids and even induction of cell death. We have previously observed that in T cells from patients with lupus, the increased mROS is capable of provoking oligomerisation of mitochondrial antiviral stimulator (MAVS) and production of type I interferon (IFN-I). mROS in SLE neutrophils also promotes the formation of neutrophil extracellular traps (NETs), which are increased in lupus and implicated in renal damage. As a result, in addition to traditional immunosuppression, more comprehensive treatments for lupus may also include non-immune therapy, such as antioxidants.

**Methods:**

Lupus-prone MRL-*lpr* mice were treated from weaning for 11 weeks with the mitochondria-targeted antioxidant, MitoQ (200 µM) in drinking water. Mice were then assessed for ROS production in neutrophils, NET formation, MAVS oligomerisation, serum IFN-I, autoantibody production and renal function.

**Results:**

MitoQ-treated mice manifested reduced neutrophil ROS and NET formation, decreased MAVS oligomerisation and serum IFN-I, and reduced immune complex formation in kidneys, despite no change in serum autoantibody.

**Conclusions:**

These findings reveal the potential utility of targeting mROS in addition to traditional immunosuppressive therapy for lupus.

## Introduction

SLE is a complex and heterogeneous autoimmune syndrome characterised by numerous abnormalities, including various autoantibodies, the appearance of unusual CD4-CD8- TCR-αβ^+^ cells, altered metabolism of lymphocytes, a type I interferon (IFN-I) gene signature in peripheral blood mononuclear cells (PBMC), increased formation of neutrophil extracellular traps (NETs), and deposition of immunoglobulins and complement at renal glomeruli.^1–10^ Each of these immune abnormalities may provoke different aspects of the disease, and each also may be driven by different environmental and genetic aberrations. Recent work has also revealed the importance of non-immune factors, such as oxidative stress, in the development of end-organ damage in SLE, shifting the paradigm of SLE pathogenesis from that of a disease provoked solely by a disturbed immune system.^1 7^ Thus, fully effectively treatment of SLE may require inhibiting pathways in addition to those targeted by traditional immunosuppressive medications.

Neutrophil activation in response to glomerular immune complexes contributes to lupus glomerulonephritis, in part through the production of mitochondrial reactive oxygen species (mROS) that can directly injure tissue through oxidation of lipids, proteins, DNA and induction of apoptosis.^11 12^ We have shown previously that immune complex activation of neutrophils can also lead to the formation of NETs, which can release oxidised genomic and mitochondrial DNA and promote the production of IFN-I.^10 12^ Furthermore, a subset of low-density granulocytes from patients with SLE has an enhanced capacity to form NETs in an mROS-dependent manner, and these structures have been observed in the kidneys of patients with lupus nephritis as well as in other lupus-affected tissues.^11 13^

We have also previously observed that T lymphocytes from patients with SLE manifest mitochondrial dysfunction as evidenced by enlarged mitochondria and elevated mROS that can induce oxidative damage.^14^ The mROS triggers the spontaneous oligomerisation of the mitochondrial antiviral stimulator (MAVS) protein and downstream IFN-I production.^15^ This was likely due in part to induction of cysteine disulfide bonding in the caspase recritment domain (CARD) of MAVS, since a C79F polymorphism of MAVS known to be associated with milder SLE^16^ did not efficiently oligomerise nor induce IFN-I with mROS.^15^ In addition, we observed that oligomerised MAVS is reversed in vitro in the presence of the mitochondrial antioxidant, MitoQ.^15^ Consequently, mROS and resulting oxidative damage emerge as possible driving forces in certain aspects of SLE, and hence represent a target for therapy.

The process that promotes the mitochondrial abnormalities in human SLE T cells is unknown. Hence, we examined whether a similar phenomenon of enlarged mitochondria and spontaneous MAVS oligomerisation might occur in any of the T-cell subsets of lupus-prone MRL-*lpr* mice. T cells in these mice accumulate in large numbers in lymphoid organs through dysregulated homeostatic proliferation that is enhanced in the absence of the death receptor, Fas (CD95).^17^ The dysregulation of T cells includes the emergence with age of an increasingly large proportion of polyclonal CD4^–^CD8^–^ TCR-αβ^+^ cells that derive from CD8^+^ precursors during homeostatic proliferation.^17^ A CD4^–^CD8^–^ TCR-αβ^+^ subset also occurs in human SLE.^3 4^ Similar to human SLE T cells, the *lpr* CD4^–^CD8^–^ TCR-αβ^+^ subset also manifested enlarged mitochondria and spontaneous MAVS oligomerisation. We thus investigated further the ability of the mitochondria-targeted antioxidant MitoQ in vivo to reverse mROS and NET formation, MAVS oligomerisation, as well as to test its therapeutic potential on lupus disease manifestations in MRL-*lpr* mice.

## Methods

### Mice

Mice were bred and housed in the Association for Assessment and Accreditation of Laboratory Animal Care International-approved animal facilities of The University of Vermont Larner College of Medicine. Original breeding pairs of MRL/MpJ-*Fas^lpr^* (MRL-*lpr*) mice were obtained from Jackson Laboratory (Bar Harbor, Maine, USA). All breeding and animal studies were conducted in accordance with the policies of The University of Vermont’s Animal Care and Use Committee.

### MitoQ treatment

Mice were weaned at 4 weeks and placed on either drinking water alone or 200 µM MitoQ (MitoQ, Auckland, NZ) in drinking water. Bottles were changed weekly. After 11 weeks of treatment, mice were euthanised and kidneys harvested for histological analysis. Brachial and axillary lymph nodes were assessed for cell number and lymphocyte subsets were determined by flow cytometry. Neutrophils were purified from bone marrow as previously described.^10^ Serum was obtained for autoantibodies, creatinine and blood urea nitrogen (BUN). Urine was obtained for creatinine measurement.

### Lymphocyte preparation

Single cell suspensions of lymph nodes were prepared in Roswell Park Memorial Institute (RPMI) 1640 (CellGro, Corning, Manassas, Virginia, USA) containing 25 mM 4-(2-hydroxyehtyl)-1-piperazineethanesulfonic acid (HEPES), 5% v/v bovine calf serum (HyClone, Logan, Utah, USA), 5×10^−5^ M β-mercaptoethanol (Sigma, St. Louis, Missouri, USA), 100 U/mL penicillin and 100 U/mL streptomycin (Life Technologies-Invitrogen; Grand Island, New York, USA) (RPMI/5% bovine calf serum (BCS)). CD8^+^ and CD4^–^CD8^–^TCRαβ^+^ T cells were isolated by negative selection. Lymph node cells were incubated with the appropriate antibodies (see below) for 30 min on ice. After washing, the cells were incubated by rocking with goat anti-rat and goat anti-mouse IgG-coated beads (Qiagen, Valencia, California, USA) for 45 min at 4°C. Antibody-coated cells were removed by magnetic depletion. To obtain CD8^+^ T cells, cell suspensions were incubated with anti-class II (3F12), anti-CD11b (M1/70), anti-NK1.1 (PK136), anti-kappa (187.1), anti-CD4 (GK1.5) and anti-CD45R (B220, RA3GB2). To isolate CD4^–^CD8^–^TCRαβ^+^ T cells, cells were incubated with anti-class II (3F12), anti-CD11b (M1/70), anti-NK1.1 (PK136), anti-kappa (187.1), anti-CD4 (GK1.5) and anti-CD8 (Tib105).

### Antibodies and flow cytometry

The following antibodies to murine cell surface proteins were purchased from BD Biosciences (San Jose, California, USA): allophycocyanin (APC)-conjugated anti-TCRβ and Pacific Blue-conjugated anti-CD45R (B220). The following antibodies were purchased from Life Technologies-Invitrogen: phycoerythrin (PE)-conjugated anti-CD44, PE-Texas Red-conjugated anti-CD4, PE Cy5.5-conjugated anti-CD8 and Pacific Orange-conjugated anti-CD45. The following antibodies were purchased from BioLegend (San Diego, California, USA): Alexa 647-conjugated anti-TCRγδ, Alexa 700-conjugated anti-CD19 and Pacific Blue-conjugated anti-CD19. Live Dead Fixable Blue was purchased from Life Technologies-Molecular Probes. Lyophilised rat IgG and hamster IgG (MP Biochemicals, Solon, Ohio, USA) were resuspended in phosphate-buffered saline (PBS) and stored at −80°C.

For direct staining, single cell suspensions were washed with cold (4°C) PBS and then incubated with Live Dead Fixable Blue Stain (Life Technologies-Molecular Probes, Eugene, Oregon, USA) in PBS for 30 min at 4°C. The cells were washed with cold PBS containing 1% w/v bovine serum albumin (BSA) fraction V (Sigma) (PBS/1% BSA) and then incubated with a mixture of rat IgG and hamster IgG (50 µg/mL each) for 30 min at 4°C. After washing, the cells were incubated with the appropriate antibodies in PBS/1% BSA, washed and fixed with freshly made 1% v/v methanol-free formaldehyde (Ted Pella, Redding, California, USA) in PBS/1% BSA. Flow cytometry was performed on an LSR II (BD Biosciences) and the data were analysed using FloJo software (Tree Star, Ashland, Oregon, USA).

### Analysis of mitochondrial morphology by transmission electron microscopy

Cells were fixed for 1 hour at 65°C in 2% paraformaldehyde and 2.5% glutaraldehyde (Polysciences, Warrington, Pennsylvania, USA) in 100 mM sodium cacodylate buffer (pH 7.2). Samples were washed in cacodylate buffer and then postfixed for 1 hour in 1% osmium tetroxide (Polysciences). Samples were then extensively rinsed in distilled H_2_O before undergoing en bloc staining for 1 hour with 1% aqueous uranyl acetate (Ted Pella). After several rinses in distilled H_2_O, the samples were dehydrated in a graded series of ethanol and then embedded in Eponate 12 resin (Ted Pella). Sections (95 nm in thickness) were cut with an Ultracut UC7 ultramicrotome (Leica Microsystems, Wetzlar, Germany), stained with uranyl acetate and lead citrate, and viewed on a JEOL 1400 transmission electron microscope (JEOL USA) equipped with an XR611 high-resolution, 11-megapixel mid-mount charge-coupled device camera (Advanced Microscopy Techniques, Woburn, Massachusetts, USA).

### Semidenaturing detergent agarose gel electrophoresis (SDD-AGE) for the detection of MAVS oligomers

SDD-AGE was performed according to a published protocol with minor modifications.^18^ Briefly, mitochondria were resuspended in sample buffer (0.5× tris-borate EDTA (TBE), 10% glycerol, 2% sodium dodecyl sulfate (SDS) and 0.0025% bromophenol blue) and loaded onto a vertical 1.5% agarose gel. After electrophoresis in running buffer (1× TBE, 0.1% SDS) for 35 min with a constant voltage of 75 V at 4°C, proteins were transferred to polyvinylidene difluoride (PVDF) membranes with a Trans-Blot Turbo Transfer System in preparation for western blot analysis. PVDF membranes were blocked in Tris-buffered saline and 5% non-fat powdered milk and analysed with MAVS-specific antibody (Santa Cruz Biotechnology, Dallas, Texas, USA). Immunoreactive proteins were visualised with horseradish peroxidase–labelled conjugates (Jackson ImmunoResearch, West Grove, Pennsylvania, USA) and developed with Clarity Western ECL Substrate (Bio-Rad, Hercules, California, USA). Chemiluminescence was detected and recorded with a Bio-Rad Chemidoc instrument. Densitometric measurements were performed in Image Lab image acquisition and analysis software (Bio-Rad).

### Metabolism analysis

Real-time analysis of extracellular acidification rates and oxygen consumption rates were measured with the XFe96 extracellular flux analyser (Agilent Technologies, Santa Clara, California, USA) according to the manufacturer’s specifications. Metabolic profiles were measured under basal conditions in non-buffered Dulbecco's Modified Eagle Medium (Sigma) containing 25 mM glucose, 2 mM L-glutamine and 1 mM sodium pyruvate, in response to 1 mM oligomycin, 0.5 mM trifluoromethoxy carbonylcyanide phenylhydrazone (FCCP) and 1 mM rotenone/1 mM antimycin. Analysis was performed with the Wave Software V.2.4 or V.2.6 (Agilent Technologies).

### Quantification of NETs and mROS in bone marrow neutrophils

The isolation of bone marrow-derived neutrophils and quantification of NETs and mROS were performed as previously described.^10^ Briefly, hindlimb marrow neutrophils were purified by Percoll gradient. Cells were seeded in a 96-well plate (2 00 000 cells/100 µL/well) in triplicates for each dye and allowed to form NETs in the presence of SYTOX (externalised DNA, 1 µM final concentration), Quant-It Picogreen (total DNA stock solution diluted 1:250) and MitoSox (200 ng/mL) (all from Thermo Fisher, Waltham, Massachusetts, USA). Fluorescence was measured at different time points for each dye, at the earliest time point 485/520 (Picogreen), 1 hour 510/580 (MitoSox) and 2 hour 486/520 (SYTOX), using a FLUOstar Omega BMG Labtech (Cary, North Caroline, USA) plate reader. Picogreen measurement was used as the initial number of cells or total DNA.

### Autoantibody quantification

Serum concentrations of autoantibodies were determined using commercially available ELISA kits (Alpha Diagnostic International, Texas, USA). Serum was diluted (1:125) in non-specific binding (NSB) buffer and the assay done following manufacturer’s instructions.

### Assessment of kidney histology and function

Renal immune complex deposition was quantified as previously described^10^ using an Alexa Fluor 594 F(ab’)2-goat antimouse IgG (Thermo Fisher) and fluorescein isothiocyanate (FITC)-antimurine C3 antibody (Immunology Consultants Laboratories, Portland, Oregon USA). Nuclei were stained with Hoechst (1:500; Life Technologies, Carlsbad, California, USA). For quantification, three random images were obtained from each stained frozen section. The images were analysed with Image J software selecting the glomerular compartment to quantify mean pixels for each fluorescence channel used.

To quantify serum creatinine and eliminate the influence of chromogens in mouse serum that interfere with the classic Jaffe method for creatinine detection, an high performance liquid chromatography assay was used as previously described.^19^ Briefly, 5 µL serum were treated with 0.5 mL acetonitrile, centrifuged at 4°C at 13 000×*g* for 20 min, and supernatants were dried by SpeedVac and resuspended in mobile phase (5 mM sodium acetate, pH 5.1). Duplicates were run on a 100×4.1 mm PRP-X200 column (Hamilton, Reno, Nevada, USA) and isocratically eluted at 2 mL/min in an Agilent 1100 System, with ultraviolet detection at 234 nm. Absolute quantitation was determined with a standard curve of 2–50 ng creatinine (r^2^=0.999).

### Statistical analysis

Statistical analyses were performed using the graphing software Prism V.7 (GraphPad Software, La Jolla, California, USA). The following statistical tests were used: paired and unpaired t-test when comparing two conditions, one-way analysis of variance (ANOVA) with Tukey’s test for correction for multiple comparisons when comparing multiple conditions and two-way ANOVA with Sidak test for correction for multiple comparisons when comparing multiple variables across multiple conditions. All data met the assumptions of the statistical tests used and variation among the compared groups was similar.

## Results

### MRL-*lpr* CD4^–^CD8^–^ TCR-αβ^+^ cells have enlarged mitochondria, increased oxygen consumption and glycolysis

**Figure 1:**
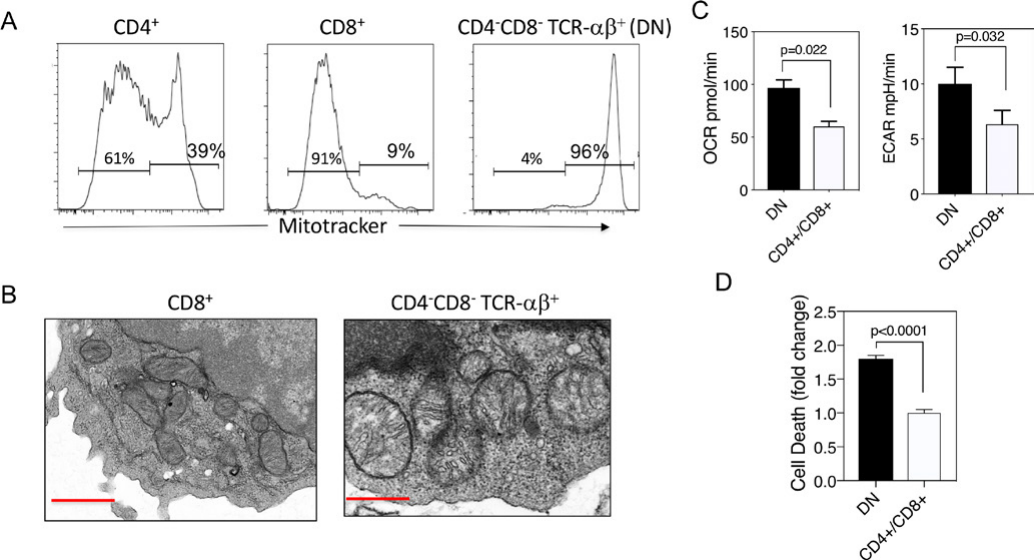
MRL-*lpr* CD4^–^CD8^–^ TCR-αβ^+^ cells have enlarged mitochondria and increased oxidative and glycolytic metabolism. (A) Lymph node cells from MRL-*lpr* mice were analysed by flow cytometry for the expression of TCR-αβ^+^, CD4, CD8 and mitochondrial mass using MitoTracker. (B) Electron micrographs (12 000×) of mitochondria from MRL-*lpr* CD8^+^ or CD4^–^CD8^–^ T cells. Red bar inset represents 500 nm. (C) OCR and ECAR for freshly isolated MRL-*lpr* CD4^+^ plus CD8^+^ T cells or CD4^–^CD8^–^ T cells determined in extracellular flux analyser. Shown are means±SEM of five replicates of each subset. (D) Spontaneous cell death following a 2-hour incubation in complete medium at 37°C. Findings and means±SEM for three experiments normalised to death rate of CD4^+^ plus CD8^+^ subset. Results are representative of three experiments. Statistical analysis was by unpaired t-test. DN, double negative; ECAR, extracellular acidification rate; OCR, oxygen consumption rate.

Our previous observations in human SLE T cells revealed that they manifest enlarged mitochondria, mROS production and spontaneous MAVS oligomerisation.^5 14 15^ We thus examined lupus-prone MRL-*lpr* mouse T cells for similar features. Initial analysis revealed that *lpr* CD8^+^ T cells, the precursors of the CD4^–^CD8^–^ TCR-αβ^+^ T cells,^17 20 21^ contained relatively low mitochondrial mass, using MitoTracker and flow cytometry, whereas the CD4^–^CD8^–^ TCR-αβ^+^ T cells had markedly higher mitochondrial mass relative to the CD8^+^ T cells (figure 1A). Further analysis by electron microscopy revealed that, similar to human SLE T cells^5 14^ the *lpr* CD4^–^CD8^–^ TCR-αβ^+^ T cells contained very large and rounded mitochondria, in contrast to the more typical elongated mitochondria of the CD8^+^ subset (figure 1B). This paralleled greater rates of oxygen consumption and glycolysis in the CD4^–^CD8^–^ TCR-αβ^+^ subset, as detected by Seahorse extracellular flux analysis (figure 1C). The increased aerobic glycolysis of CD4^–^CD8^–^ TCR-αβ^+^ T cells is consistent with the known rapid proliferation by this subset in vivo.^22^ This is paralleled by increased spontaneous cell death of the CD4^–^CD8^–^ TCR-αβ^+^ T cells compared with the CD4^+^ and CD8^+^ T cell subsets (figure 1D), consistent with previous observations that high levels of glycolysis in T cells, including CD4^–^CD8^–^ TCR-αβ^+^ T cells, drives high levels of active caspase-3, rendering them prone to cell death.^23 24^ Such increased cell death could contribute to the inflammatory response in these mice.

**Figure 2:**
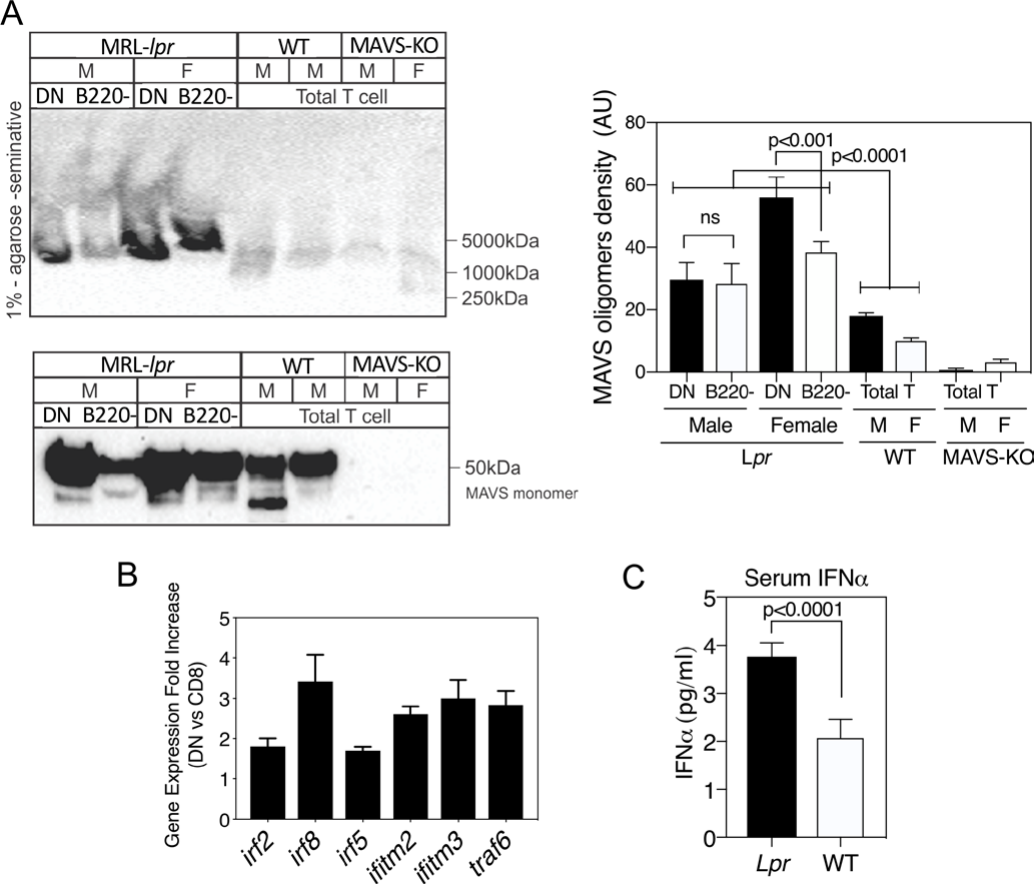
MRL-*lpr* CD4^–^CD8^–^ TCR-αβ^+^ cells manifest spontaneously oligomerised mitochondrial antiviral stimulator (MAVS) and increased expression of type I interferon (IFN-I)-stimulated genes (ISG) and serum IFN-I. (A) Lymph node cells from MRL-*lpr* mice were separated into CD4^–^CD8^–^ TCR-αβ^+^ (double-negative (DN)) and CD4^+^ plus CD8^+^ (B220^−^) subsets and cell lysates prepared. Comparisons were made with lymph node cells from MRL^+/+^ wild-type (WT) or MAVS-knockout (KO) mice. Lysates were subjected to 1% agarose gels (upper panel) to selectively visualise MAVS oligomers, or SDS-PAGE (sodium dodecyl sulfate–polyacrylamide gel electrophoresis; lower panel) to visualise total MAVS monomer levels. (B) Fold increase in RNA expression for several ISG comparing female MRL-*lpr* DN to naive CD8^+^CD44^low^ cells of the same mice. RNA was hybridised to Affymetrix GeneChip Mouse 430 2.0. Results are from five untreated 10-week-old mice per experiment and performed three times. (C) Serum levels of IFN-I in female MRL^+/+^ WT and MRL-*lpr* mice. Shown are the mean±SEM of five mice of each type. Statistical analysis was by unpaired t-test. Results were very similar in three experiments. F, female; M, male.

Given these parallels between T cells from human SLE and the CD4^–^CD8^–^ TCR-αβ^+^ T cells of MRL-*lpr* mice, we examined whether they also manifested evidence of spontaneous MAVS oligomerisation and activation of IFN-I genes. Indeed, the CD4^–^CD8^–^ TCR-αβ^+^ (B220^+^) subset contained MAVS oligomers, which were diminished in the CD4^+^ and CD8^+^ (B220^−^) fraction and non-existent in wild-type mice (figure 2A). This paralleled the upregulation of several IFN-I-stimulated genes in the CD4^–^CD8^–^ TCR-αβ^+^ T cells compared with the CD8^+^ precursors (figure 2B). Consistent with these findings, MRL-*lpr* mice had higher levels of serum IFNα compared with wild-type mice (figure 2C). Collectively, these findings suggest that mROS may drive MAVS oligomerisation in *lpr* CD4^–^CD8^–^ TCR-αβ^+^ T cells as it does in human SLE. We thus considered that mROS and possible oxidative damage might also drive some of the disease manifestations in MRL-*lpr* mice, and elected to treat the mice in vivo with the mitochondria-targeted antioxidant MitoQ.

### In vivo MitoQ treatment of MRL-*lpr* mice reduces ROS production and NET formation by neutrophils

Increased production of mROS and NET formation has been observed in neutrophils of patients with SLE.^10 11^ As NET formation in human lupus is, at least in part, driven by mROS production, we initially examined whether the mitochondria-targeted antioxidant, MitoQ, would reduce neutrophil mROS and NET formation. MitoQ contains an antioxidant, ubiquinonol, coupled to a triphenylphosphonium moiety containing three phenyl groups to promote membrane permeabilisation, combined with a central positively charged phosphorous that draws the compound toward negative charges. As mitochondria are about 150–170 mV negative compared with the cytosol, which is itself a further 30–60 mV negative compared with the extracellular environment, Mito compounds are concentrated 500–1000× in mitochondria.^25–27^

**Figure 3:**
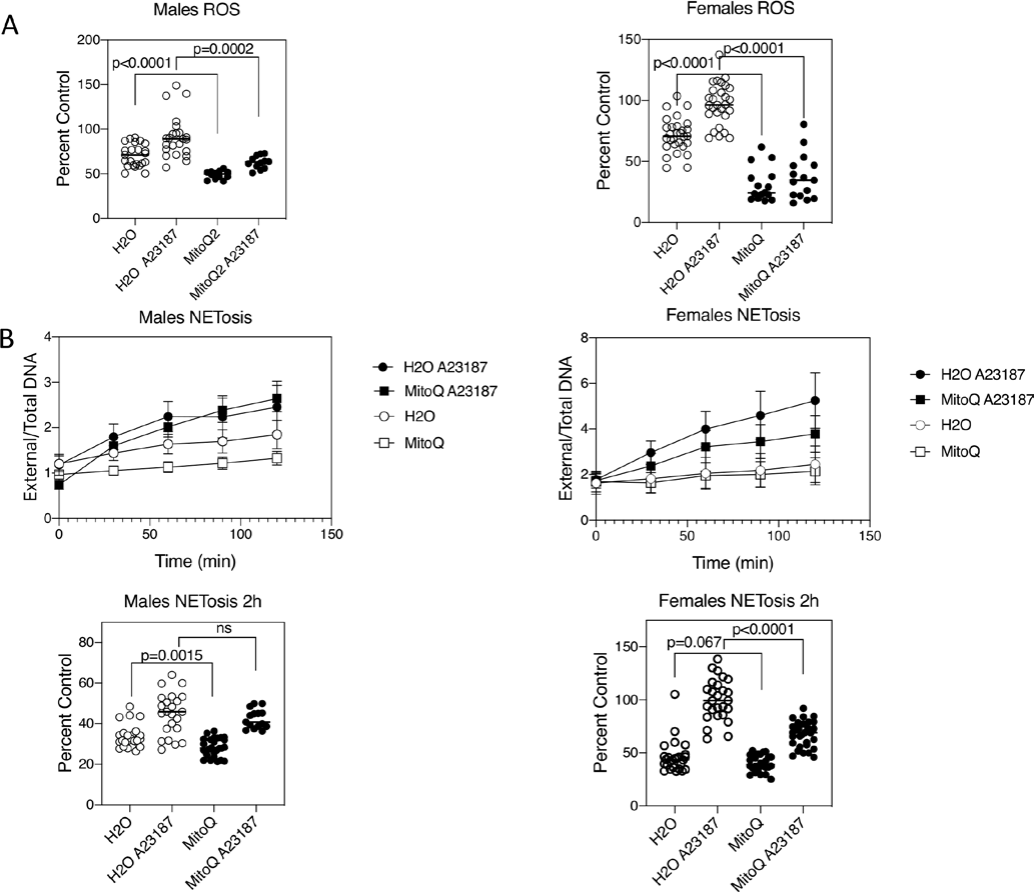
In vivo MitoQ treatment reduces levels of reactive oxygen species (ROS) and neutrophil extracellular trap (NET) formation in neutrophils. Mice (eight mice per treatment group) were administered either drinking water alone, or water containing MitoQ (200 µM) for 11 weeks following weaning. Neutrophils were purified from bone marrow and assayed for (A) ROS production and (B) NET formation either spontaneously or following activation with A23187. Per cent control refers to the comparison of each value to the mean of female neutrophils with A23187 activation. Shown are points for individual mice and their mean for three experiments. Statistical analysis was unpaired t-test. ns, non-significant.

Mice were administered MitoQ (200 µM) in their drinking water beginning at 4 weeks of age and continued for 11 weeks. No adverse effects of MitoQ (eg, weight, development) were observed in mice at this dose, consistent with other studies.^27–30^ mROS production in neutrophils was initially examined by MitoSox fluorescence plate assay. This revealed a reduction in mROS production in MitoQ-treated mice, both spontaneously and following mROS stimulation with the calcium ionophore, A23187 (figure 3A). These findings were observed for both male and female MRL-*lpr* mice. Consistent with these findings, NET formation was also reduced with MitoQ treatment. In male mice, this was statistically significant for spontaneous NET formation, and in females this was the case following A23187 stimulation (figure 3B).

**Figure 4:**
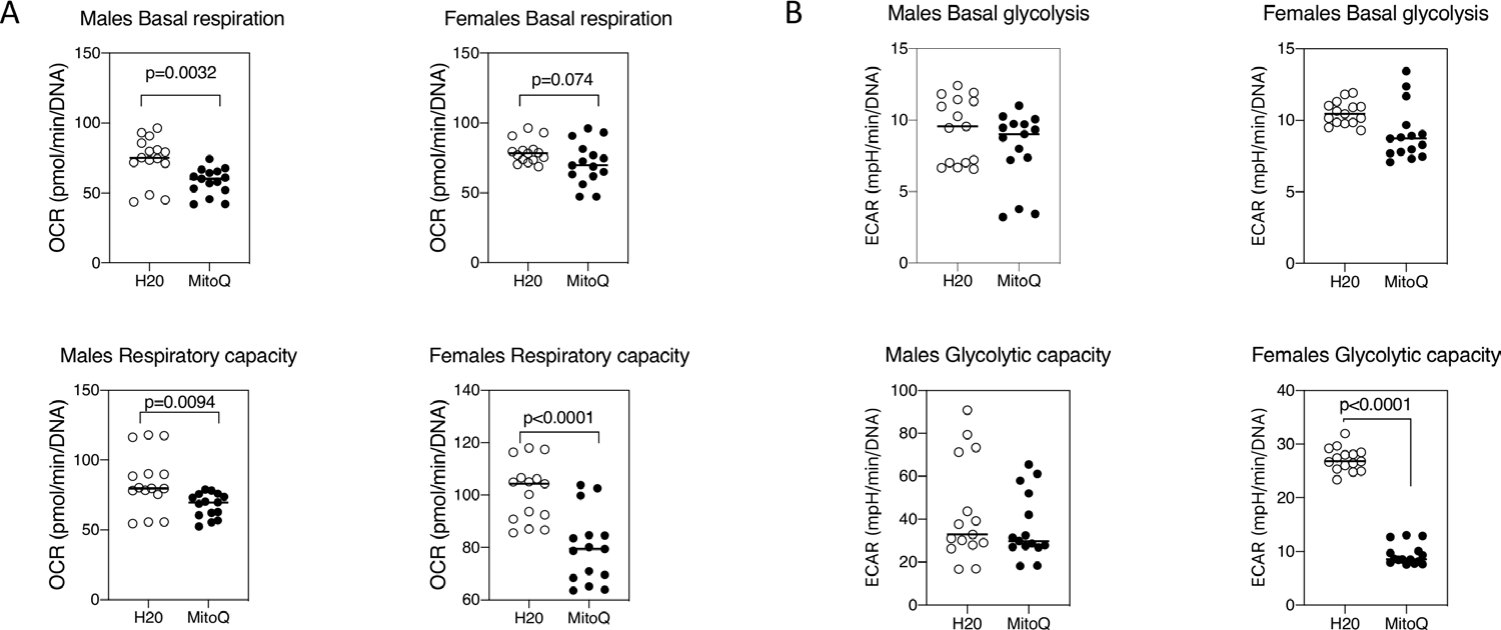
Reduced oxygen consumption and glycolysis following in vivo MitoQ treatment. Extracellular flux analysis for (A) oxygen consumption rate (OCR) and (B) glycolysis as measured by extracellular acidification rate (ECAR) for neutrophils from mice (six mice per group, two experiments) administered water alone or MitoQ (200 µM) in drinking water. Shown are mean and statistical analysis by unpaired t-test. Results are representative of three experiments.

Further analysis of metabolism in the neutrophils revealed reduced oxygen consumption with MitoQ, both at basal levels and following stimulation of respiration with the mitochondrial uncoupler FCCP (respiratory capacity) (figure 4A). In contrast, glycolysis was largely unchanged, except in the case of female mice where the glycolytic capacity, following addition of ATP synthetase inhibitor oligomycin, was decreased with MitoQ (figure 4B). These results indicate that oral MitoQ modulates lupus neutrophil immunometabolism and reduces their ability to form NETs.

### MitoQ in vivo reduces MAVS oligomerisation and serum IFN-I

**Figure 5:**
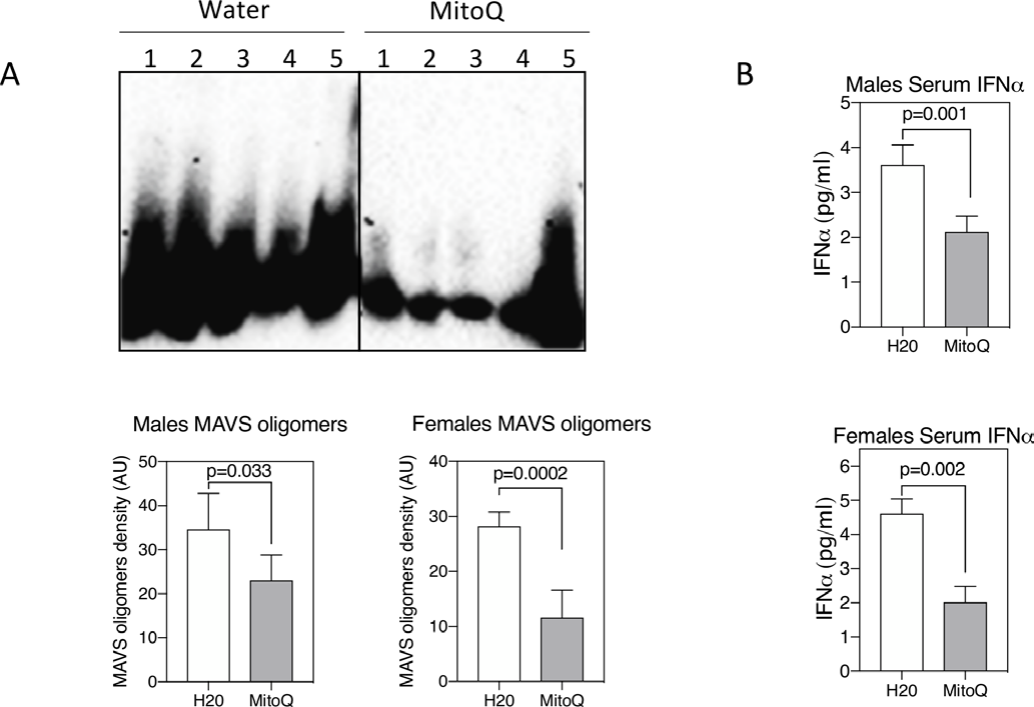
Reduced mitochondrial antiviral stimulator (MAVS) oligomerisation and serum interferon (IFN)-β with MitoQ. (A) MAVS oligomers were assessed by western blot from non-denaturing agarose gels as described in the Materials and methods section. Shown are results from five mice per treatment group. Graphs indicate density of MAVS oligomers. (B) Serum IFNα as measured by ELISA. Shown are mean±SEM and statistical analysis by unpaired t-test. Results are representative of two experiments.

**Figure 6:**
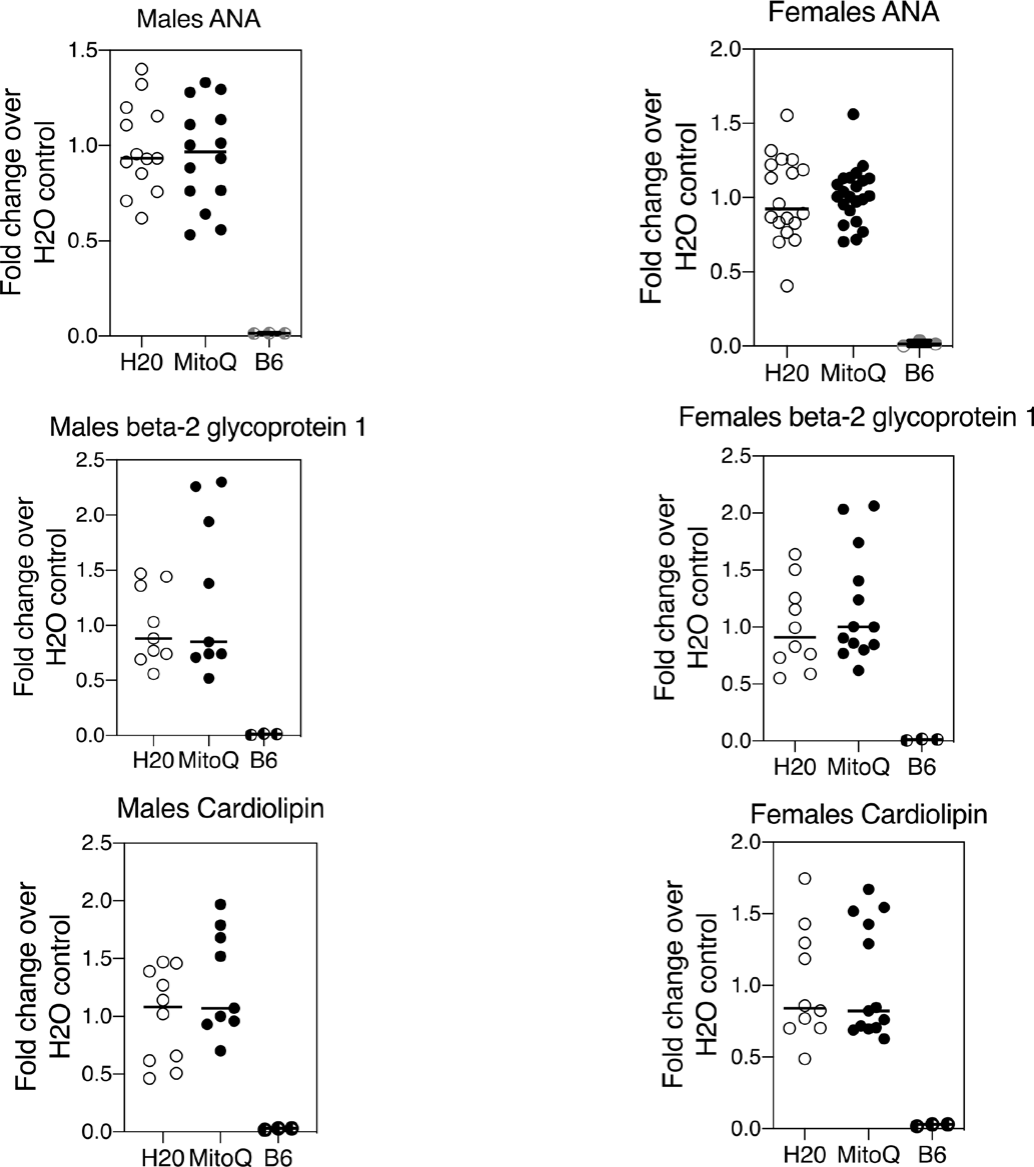
MitoQ does not alter autoantibody titres in MRL*-lpr* mice. MRL-*lpr* mice received water only or water containing MitoQ for 11 weeks. Serum autoantibody titres were measured by ELISA. Results shown are the combined findings of three experiments normalised to the water-only controls. Numbers of MRL-*lpr* mice in each treatment group (n=H_2_0/MitoQ) were: males ANA (n=13/14), males beta-2 glycoprotein 1 (n=9/9), males cardiolipin (n=10/9), females ANA (n=18/22), females beta-2 glycoprotein 1 (n=10/13), and females cardiolipin (n=10/13).

CD4^–^CD8^–^ TCR-αβ^+^ T cells purified from MitoQ-treated mice revealed reduced levels of MAVS oligomerisation compared with mice receiving water only (figure 5A). This was paralleled by a reduction in serum IFNα (figure 5B). In contrast to the reduction of NET formation and MAVS oligomerisation, MitoQ treatment did not affect lymph node total cell numbers, the distribution of T-cell subsets, including CD4^–^CD8^–^ TCR-αβ^+^ T cells, or B cells (not shown), nor the titres of serum autoantibodies (figure 6) in male or female MRL-*lpr* mice. This was somewhat anticipated as MitoQ was not expected to alter events believed to be upstream of mROS production.

### Reduced renal injury with MitoQ

**Figure 7:**
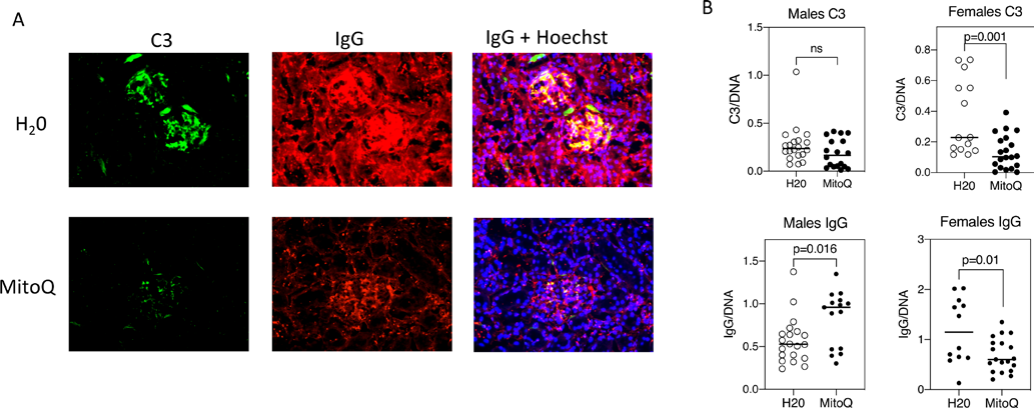
MitoQ reduces kidney immune complexes in female mice. Kidneys were stained for C3 (green), IgG (red) and nuclei by Hoechst stain (blue) and imaged by fluorescence microscopy. The glomerular-associated fluorescence was analysed by pixel quantification in each fluorescence channel using Image J. An example is shown in (A) and summaries all mice analysed is shown in (B).

**Figure 8:**
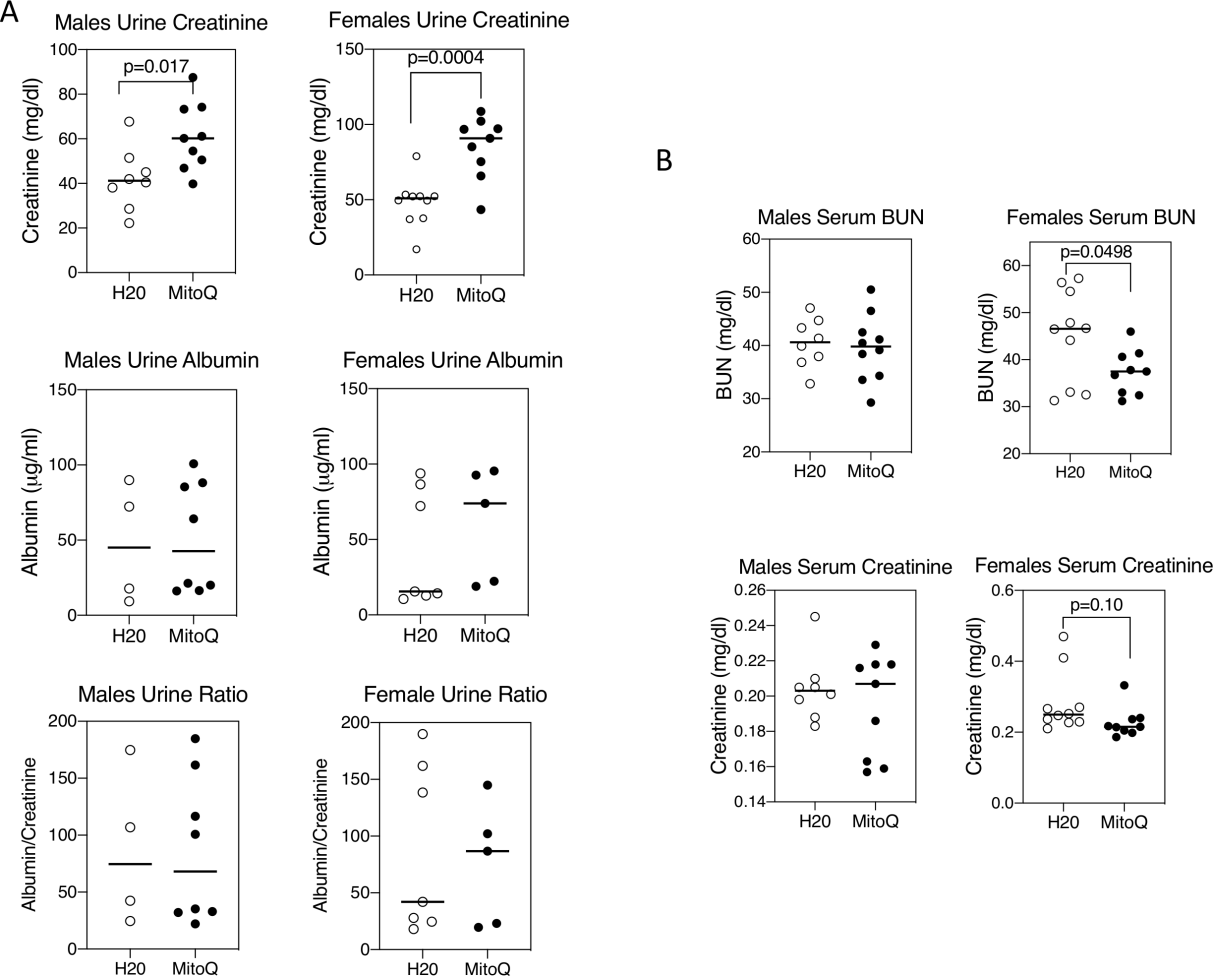
MitoQ improves renal function in female mice. Urine and serum were collected from mice at the end of 11 weeks on water alone or water containing MitoQ. (A) Urine creatinine and albumin concentrations were measured as detailed in the Material and methods section. (B) Serum blood urea nitrogen (BUN) and creatinine concentrations were determined as in the Materials and methods section. Results show mean and individual values for 8–10 mice in each treatment group and of each sex. Statistical analysis was unpaired t-test. Results are representative of two experiments.

MRL-*lpr* mice develop immune complex-mediated kidney damage with age.^31 32^ MitoQ treatment of female mice caused a reduction of the glomerular deposition of both C3 and IgG (figure 7). This was less apparent in male mice, but male control mice had less C3 and IgG deposition than female control mice, so there was a narrower window in male mice in which to observe a difference with MitoQ. These findings were paralleled by increased urine excretion of creatinine, although without a change in urine albumin (figure 8A) and was also accompanied by a reduction in serum BUN and creatinine in female mice (figure 8B).

## Discussion

The current findings suggest that inhibiting mROS and oxidative stress can mitigate certain aspects of autoimmune disease and organ damage in lupus-prone MRL-*lpr* mice. It has been appreciated for some time that T cells and neutrophils in patients with SLE manifest increased levels of ROS,^5 6^ but the consequences of this on other molecular abnormalities, immune dysregulation or disease manifestations remain largely unexplored. The current findings reveal that the mitochondria-targeted antioxidant MitoQ can reduce oligomerisation of MAVS and serum IFN-I, mitigate glomerular immune complex formation and help preserve renal function.

The IFN-I gene signature in PBMC is one of the hallmarks of SLE,^8 9^ yet the mechanism is unclear. The IFN-I signature may be driven in part through engulfment of dead cells by macrophages and dendritic cells.^33^ The increased rate of cell death of neutrophils in patients with SLE, as well as the increased cell death of CD4^–^CD8^–^ TCR-αβ^+^ T cells in MRL-*lpr* mice and human SLE T cells, may both contribute to the source of dead cells. The released nucleic acids can trigger both ROS and IFN-I production. This is clear from genetic deficiencies in DNA clearance (eg, three prime repair exonuclease (TREX)) which lead to interferonopathies.^34 35^

Another source of ROS in human SLE comes from T cells, some of which manifest enlarged mitochondria.^5 14^ We have shown previously that ROS can induce MAVS oligomerisation and IFN-I production in different cell types.^15^ We now observe that, similar to human SLE, mitochondrial enlargement and spontaneous MAVS oligomerisation arises in the MRL-*lpr* CD4^–^CD8^–^ TCR-αβ^+^ subset. This has been attributed to an imbalance of mitochondrial biogenesis^36^ and turnover by mitophagy.^37^ Specifically, our previous studies of SLE T cells have demonstrated human retrovirus endogenous sequence (HRES)-1/ras-related protein 4 (RAB4)-mediated depletion of dynamin related protein 1 (Drp1), a mediator of mitochondrial fission.^36^ The accumulation of mitochondria in lupus T cells is sensitive to mTOR blockade, with clinical benefit in patients^38^ and mice^39^ with SLE.

The CD4^–^CD8^–^ TCR-αβ^+^ subset, which also occurs in human SLE, including inflamed kidneys,^40^ arises from recurrent homeostatic proliferation of CD8^+^ precursor T cells.^17 41^ CD4^–^CD8^–^ TCR-αβ^+^ T cells also derive from antigen-specific CD8^+^ T cells in wild-type mice only when the antigen is presented as a self-antigen.^42^ Gene expression profiling of these wild-type derived CD4^–^CD8^–^ TCR-αβ^+^ T cells has revealed upregulation of genes for programmed cell death 1 (PD-1), interleukin 17, IFNγ, C-X-C Motif Chemokine Ligand 2 (CXCL2) and downregulation of CD127,^43^ exactly the same pattern observed in *lpr* CD4^–^CD8^–^ TCR-αβ^+^ T cells.^22^ Thus, these unusual T cells are not unique to Fas-deficient *lpr* mice. These findings, in addition to our previous observations that T-cell homeostatic proliferation also leads to the upregulation of genes involved with cytolysis and inflammation^22^ adds further importance why it is critical to regulate the homeostatic expansion of T cells with Fas. In addition to these gene expression changes, our findings also suggest that part of the programme of dysregulated T-cell homeostatic proliferation in *lpr* mice includes mitochondrial enlargement leading to mROS production and MAVS oligomerisation.

MitoQ has shown therapeutic potential in a variety of conditions in which mROS has been implicated. These include animal models of Alzheimer’s disease,^29^ liver fibrosis,^30^ NLR Family Pyrin Domain Containing 3 (NLRP3) inflammasome-mediated colitis,^28^ cardiac ischaemia–reperfusion^44^ and the metabolic syndrome in Apolipoprotein E (ApoE)^-/-^ mice.^27^ In human trials, MitoQ prevented inflammatory damage in a phase II study in hepatitis C,^45^ showed no benefit in Parkinson’s disease but was well tolerated^46^ and improved vascular function in older adults.^47^ Oxidative stress could be functioning at several levels in SLE, including induction of MAVS oligomerisation^15^ and oxidised mitochondrial DNA release during NET formation,^10^ each contributing to the activation of the IFN-I pathway. In addition, immune complex and complement deposition in renal glomeruli induces oxidative stress in renal epithelium.^10^ Thus, MitoQ may function at several points in reducing oxidative damage in the pathogenesis of SLE.

From our current understanding that MitoQ reduces mROS and oxidative stress and hence events downstream of mROS generation, we would not have anticipated MitoQ to affect events upstream of mROS production. These might include the development of lymphadenopathy of MRL-*lpr* mice, the generation of CD4^–^CD8^–^ TCR-αβ^+^ T cells and the production of autoantibodies, which may result from the absence of cell death occurring during homeostatic proliferation of lymphocytes.^17 48^ Consequently, the upregulation of genes during T-cell homeostatic proliferation involved with cytolysis and inflammation, such as granzyme B, perforin and Fas-ligand,^22^ is likely to still contribute to inflammation during MitoQ therapy. Thus, fully effective therapy for SLE may require a combination of traditional immunosuppression with non-immune antioxidant therapy.
