# Correction: Targeting mitochondrial oxidative stress with MitoQ reduces NET formation and kidney disease in lupus-prone MRL-lpr mice

**DOI:** 10.1136/lupus-2020-000387corr1

**Published:** 2020-04-27

Fortner KA, Blanco LP, Buskiewicz I, *et al*. Targeting mitochondrial oxidative stress with MitoQ reduces NET formation and kidney disease in lupus-prone MRL-lpr mice. Lupus Sci Med 2020;7:e000387.

The published version misspelled co-author’s name as Andreas Perl. The correct name should be Andras Perl.

